# Metabolomic changes related to airway inflammation, asthma pathogenesis and systemic activity following inhaled fluticasone furoate/vilanterol: a randomized controlled trial

**DOI:** 10.1186/s12931-022-02164-w

**Published:** 2022-09-20

**Authors:** Peter Daley-Yates, Brian Keppler, Amanda Baines, George Bardsley, James Fingleton

**Affiliations:** 1 Respiratory Clinical Development, GSK Research and Development, Stockley Park West, 1-3 Ironbridge Road, Uxbridge, Middlesex UB11 1BT UK; 2 Metabolon Inc., 617 Davis Drive, Suite 100, Morrisville, NC 27560 USA; 3 Medicines Development Centre, GSK Research and Development, Stevenage, UK; 4 Tauranga Hospital, 829 Cameron Road, Tauranga South, Tauranga, 3112 New Zealand; 5 Medical Research Institute of New Zealand, Wellington, New Zealand

**Keywords:** Metabolomics, Asthma, Inhaled corticosteroid, Long-acting β_2_-agonist, Clinical trial, Fluticasone furoate, Vilanterol

## Abstract

**Background:**

Fluticasone furoate/vilanterol trifenatate (FF/VI) is an inhaled therapy for the treatment of asthma, with a prolonged duration of anti-inflammatory and bronchodilatory action. This study investigated the global metabolomic and lipidomic profile following treatment with FF/VI or placebo and assessed whether changes correlated with exhaled nitric oxide levels as a measure of airway inflammation.

**Methods:**

This was a single-center, randomized, double-blind, placebo-controlled, two-period, crossover, repeat-dose study. Adults with asthma (forced expiratory volume in 1 s ≥ 60% predicted; fraction of exhaled nitric oxide [FeNO] > 40 parts per billion) received once-daily FF/VI 100 µg/25 µg or placebo for 14 days, followed by a 21-day washout period. Serum samples were taken at pre-dose (T1), and 15 and 21 days (T2 and T3, respectively) post dose in each period. The metabolomic and lipidomic profiles were analyzed by liquid chromatography with tandem mass spectrometry and polar liquid chromatography platforms, and ions were matched to a library of standards for metabolite identification and quantification. FeNO values at each timepoint were evaluated for correlations with the biochemical data.

**Results:**

Of 27 randomized participants (mean age 24.5 years, 63% male), 26 provided serum samples for metabolomic analysis. A total of 1969 metabolites were identified, 1634 of which corresponded to a named structure in a reference library. Treatment-related changes in the metabolome were generally subtle, with a modest increase in metabolite perturbations across timepoints. The percentage of metabolites with significant changes (p < 0.05 for all) (increases↑/decreases↓) versus placebo were: 2.1% (1.1%↑/1.0%↓), 6.7% (0.46%↑/6.2%↓) and 11.8% (0.86%↑/10.9%↓) at T1, T2 and T3, respectively. Treatment with FF/VI reduced FeNO levels by 60%, whereas the systemic intermediates involved in NO biosynthesis remained unaffected. Evidence of systemic anti-inflammatory activity was seen in complex lipid pathways, suggesting reduced phospholipase-A2 activity, but without downstream impact on free fatty acids or inflammatory mediators. Consistent with the pathogenesis of asthma, there was evidence of higher fatty acid β-oxidation and lower glycolysis in the placebo arm; this pattern was reversed in the treatment arm.

**Conclusions:**

Despite the prolonged airway anti-inflammatory action of FF/VI, this was accompanied by only subtle systemic metabolomic and lipidomic changes.

*Trial registration* Prospectively registered on ClinicalTrials.gov registry number NCT02712047

**Supplementary Information:**

The online version contains supplementary material available at 10.1186/s12931-022-02164-w.

## Background

Asthma is a chronic, non-communicable respiratory disease, characterized by airway inflammation, variable symptoms and expiratory airflow limitation [1]. It is associated with multiple clinical phenotypes and inflammatory endotypes, driven by heterogenous inflammatory mechanisms [2]. The Global Initiative for Asthma recommends that inhaled corticosteroids (ICS) are the mainstay of treatment, with additional therapies including long-acting β_2_-agonists (LABA) added on in a “step-up”/“step-down” approach to achieve good control of symptoms and exacerbations, whilst attempting to find the patient’s minimum effective level of treatment [1].

**Fig. 1:**
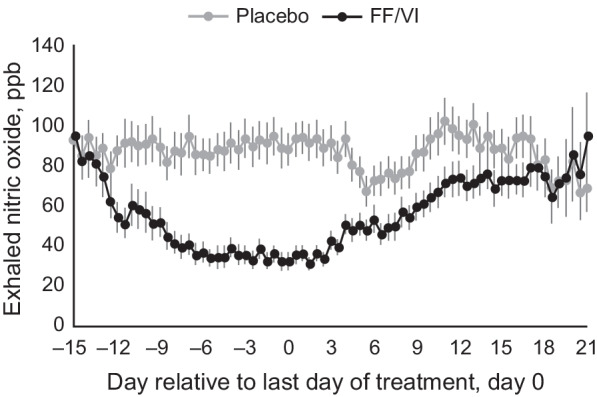
Time-course of anti-inflammatory activity of FF/VI. Figure reproduced with permission from Bardsley et al. Respir Res. 2018;19(1):133. Mean exhaled nitric oxide (ppb), during treatment and after cessation of treatment, plotted over time, for placebo (grey line) and FF/VI (black line). Error bars denote the standard error. FF/VI, fluticasone furoate/vilanterol trifenatate; ppb, parts per billion

Fluticasone furoate (FF) is an enhanced-affinity ICS with potent anti-inflammatory action and greater lung retention than other ICS therapies [3, 4]. Vilanterol trifenatate (VI) is a selective LABA which induces rapid and prolonged bronchodilation over a 24 h period [4]. FF/VI 100 µg/25 µg is an ICS/LABA combination therapy licensed for the once-daily treatment of adults and adolescents with asthma aged ≥ 12 years, and in adults with moderate-to-very severe chronic obstructive pulmonary disease who experience persistent exacerbations despite ongoing maintenance therapy. FF/VI has demonstrated a prolonged duration of bronchodilation and anti-inflammatory action in previous clinical trials of adults with asthma [4–6]. Braithwaite et al. found that bronchodilation was maintained for 72 h following a single dose of FF/VI, with clinically significant bronchodilation persisting for ≥ 48 h post dose [5]. Furthermore, Bardsley et al. [6] reported that the onset of anti-inflammatory action, as measured by a reduction in fraction of exhaled nitric oxide (FeNO) levels, occurred at 72 h post dose, with a maximum reduction 14 days post dose (Fig. 1). Anti-inflammatory effects were also maintained for 18 days following cessation of treatment [6].

The heterogeneity of asthma is increasingly highlighted by ongoing research, with underlying disease mechanisms poorly understood [2, 7, 8]. Metabolomics, the systematic analysis of functional metabolites in a given biological system, is a novel approach which is being increasingly explored to improve understanding of biomarkers and pathophysiological mechanisms that underlie various asthma phenotypes [2, 7, 8]. In addition, metabolomics is an alternative approach to assess the systemic effects of corticosteroids; conventional approaches typically rely on hypothalamic–pituitary–adrenal axis studies or long-term studies to monitor the effects on target organs [2, 7, 8]. Metabolic changes in asthma and those associated with corticosteroid treatment have been previously documented [9]. Loueiro et al. [9] documented that metabolites related to lipid peroxidation levels were able to predict disease severity, lung function, FeNO and blood eosinophils in 40 non-obese participants with asthma. A metabolomic analysis by Reinke et al. [2] reported similar results, with 15 metabolites found to be significantly dysregulated in asthma compared with healthy controls, and metabolic profiles differed between asthma severities. Furthermore, six metabolites were significantly correlated with ICS dose, although there was no observed significant interaction between ICS dose and metabolite levels [2]. Bordag et al. [10] also highlighted a similar effect of corticosteroids on the metabolome, with single-dose dexamethasone treatment in healthy adults resulting in a significant change in 150 metabolites involved in metabolic pathways known to be associated with the manifestation of systemic side effects.

This study was an exploratory secondary biomarker analysis from a primary study of the anti-inflammatory properties of FF/VI [6]. It investigated the global metabolomic and lipidomic profile of inhaled FF/VI in human serum samples from adults with asthma. Since ICS treatment is known to reduce airway inflammation, as measured by FeNO [6], we sought to identify systemic biomarkers that correlate with this treatment response. Although the focus of this study is the topical anti-inflammatory action of FF and its relationship to any systemic activity, we also assess the effects of VI, with which FF is administered in combination therapy.

There were three key objectives:

- (1) To assess whether 14 days of treatment with FF/VI was associated with any changes in the serum metabolomic and lipidomic profile.
- (2) To assess whether any metabolomic changes were correlated with airway inflammation as assessed via FeNO, either at baseline or following FF/VI treatment.
- (3) To identify any evidence for systemic bioactivity of FF/VI following 14 days of treatment, particularly on the known pathways for the systemic effects of corticosteroids.

## Methods

### Study design and participants

**Fig. 2:**
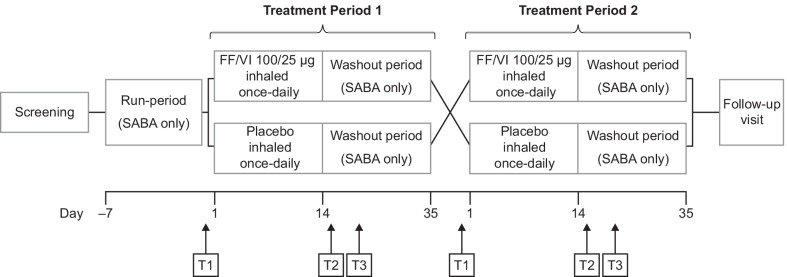
Study design. FF/VI, fluticasone furoate/vilanterol trifenatate; SABA, short-acting β_2_-agonist; T1, baseline; T2, Day 15; T3, Day 21

This was a single-center, randomized, double-blind, placebo-controlled, two-period, crossover, repeat-dose study carried out between April 2016 and February 2017, by the Medical Research Institute of New Zealand in Wellington, New Zealand (NCT02712047). The methods, including trial design and participant inclusion/exclusion criteria, have been previously described [6]. In brief, adults aged 18–65 years with asthma (forced expiratory volume in 1 s [FEV_1_] ≥ 60% predicted, reversible airway disease and FeNO > 40 parts per billion [ppb]), were randomly assigned in a 1:1 ratio to receive once-daily inhaled placebo or once-daily FF/VI 100/25 µg. The primary endpoint was change from baseline FeNO over time following the cessation of repeat dose treatment with FF/VI. Secondary endpoints included measurement of peak expiratory flow during treatment and following cessation of repeat dose treatment with FF/VI, and measurement of FEV_1_ pre-treatment and twice daily for 5 days after cessation of repeat dose treatment with FF/VI, with a final FEV_1_ reading on Day 7. Exploratory endpoints included metabolomics which are being reported separately in this article. Participants were assigned to one of two treatment sequences (AB or BA, where A was placebo, and B was FF/VI). Each treatment arm of the study had a 7-day run in period where participants received a short-acting β_2_-agonist (SABA) only; a 14-day treatment period during which participants received their allocated study medication; and a 21-day washout period where participants received SABA only. Patients were permitted to use SABA in addition to study medication in either arm, but SABA were withheld for 6 h prior to study assessments. Up to 28 days elapsed between treatment arms. Participants attended a follow-up visit following completion of both treatment arms. Serum samples were collected from all participants pre-dose (baseline, T1) and on Days 15 (post-treatment, T2) and 21 (washout, T3) for each treatment period. The study design is shown in Fig. 2.

All participants signed written informed consent prior to any study procedures and prospective ethics approval for this study was obtained from the Health and Disability Ethics Committees, New Zealand (Reference 16/STH/13).

### Sample collection

Serum samples were collected from all participants at T1, T2 and T3 for each treatment period. Samples were collected at approximately the same time of day from each participant. Upon receipt, samples were inventoried and immediately stored at – 80 °C.

Global untargeted biochemical profiling to measure metabolomic profiles was performed using Metabolon’s discovery HD4 metabolomics platform (Metabolon, North Carolina, United States). Serum samples were extracted and split into equal parts for analysis using various liquid chromatography/mass spectrometry positive, negative and polar methods which constitute the ultrahigh performance liquid chromatography-tandem mass spectroscopy platform.

Proprietary software was used to match ions to an in-house library of standards for fully annotated, Tier 1 (according to the Metabolomics Society Initiative) metabolite identification and for metabolite quantification by peak area integration (please see Additional file 1: Supplementary methods for further detail on data extraction and compound identification). Targeted quantitative lipidomic analysis was performed on the same serum samples using Metabolon’s Complex Lipid Panel (CLP). The analytical technology employed to achieve the necessary coverage, quantitation and specificity includes flow injection analysis, differential mobility separation and multiple reaction monitoring. The CLP quantifies the absolute concentration of each monitored lipid using isotopically labeled internal standards, which are included at known concentrations during the preparation of each sample.

FeNO values were collected for each participant at each timepoint as described previously [6], and assessed for statistical correlation with the biochemical data.

Further details on methodology are located within Additional file 1: Supplementary methods.

### Statistical analysis

The study sample size determination was based on the primary endpoint of FeNO and is described elsewhere [6]. Initial application of a crossover model to the data concluded there was no treatment sequence effect between populations. Statistical analysis was subsequently performed using a two-way repeated-measures analysis of variance (ANOVA) model with Treatment and Day as main effects, and which excluded sequence effects. Crossover, random forest (RF) and principal component analyses (PCA) were also used to analyze the data. Q-values were calculated using the False Discovery Rate method, which provides an estimate of the proportion of false discoveries for compounds where p < 0.05.

For all analyses, missing values, if any, were imputed with the observed minimum for each compound. All statistical analyses were performed on natural log-transformed data.

### Safety

Safety was monitored as previously described by Bardsley et al*.* [6].

## Results

### Baseline characteristics

As previously detailed by Bardsley et al. [6], 32 potential participants were screened for inclusion in the study, and 27 participants successfully completed both study periods. Of these, 26 provided serum samples. Baseline characteristics have been previously reported for the 27 participants who completed both study periods, with all reporting mild to moderate airflow obstruction at baseline (mean [standard deviation] FEV_1_ % predicted 87.7 [10.6]). Median baseline FeNO and blood eosinophils were 87 ppb (range 42–212) and 0.33 × 10^9^/L (0.18–1.18), respectively (Table 1) [6].

**Table 1:** Baseline characteristics of randomized participants [6]

Characteristic	Study population^a^ (N = 27)
Age, years, mean (SD)	24.5 (8.1)
Sex, male, n (%)	17 (63)
Ethnic origin, n (%)	
Māori	4 (15)
New Zealand European	20 (74)
Other	3 (11)
History of allergic rhinitis, n (%)	14 (51.9)
BMI, kg/m^2^, mean (SD)	24.6 (3.5)
FEV_1_^b^, % predicted, mean (SD)	87.7 (10.6)
PEF, L/min, mean (SD)	443 (99)
FeNO, ppb, median (range)	87 (42–212)
Blood eosinophil count, × 10^9^/L, median (range)	0.33 (0.18–1.18)
Blood eosinophil count ≥ 0.27 × 10^9^/L, n (%)	24 (88.9)
Serum periostin, ng/ml, median (range)	187.5 (109.4–442.5)

BMI, body mass index; FeNO, fraction of exhaled nitric oxide; FEV_1_, forced expiratory volume in 1 s; PEF, peak expiratory flow; ppb, parts per billion; SD, standard deviation

^a^Includes all participants who were randomized to primary analysis; 26 participants provided serum samples. ^b^Before bronchodilation

### Systemic metabolic effect of treatments

This study detected 1969 total metabolites (please see Additional file 2 for the full list): 1634 of these corresponded to a named structure in Metabolon’s reference library, and the remaining 335 represented a distinct chemical entity but did not match a named structure in the reference library. There was a subtle systemic metabolic effect with active treatment compared with placebo (p ≤ 0.05) for baseline (T1) and post-treatment and washout days (T2 and T3, respectively); 5% changes would be expected by random chance alone. The percentage of metabolites with significant differences (p < 0.05) (increases↑/decreases↓) between the treatment group and comparator placebo group samples, based on repeated-measures ANOVA at each timepoint, were: 2.1% (1.1%↑/1.0%↓), 6.7% (0.46%↑/6.2%↓) and 11.8% (0.86%↑/10.9%↓) at T1, T2 and T3, respectively (Table 2). Whilst there was very little biochemical discrepancy between placebo and FF/VI treatment groups at baseline (T1), the post-treatment (T2) and washout (T3) timepoints displayed subtle changes in metabolite perturbations.

**Table 2:** Statistical summary of significantly altered biochemicals

Repeated-measures ANOVA	T1 FF/VI vs placebo	T2 FF/VI vs placebo	T3 FF/VI vs placebo
Total number of biochemicals with p ≤ 0.05	42	132	232
Biochemicals (increase ¦ decrease)	22 ¦ 20	9 ¦ 123	17 ¦ 215
% significant	2	7	12

ANOVA, analysis of variance; FF/VI, fluticasone furoate/vilanterol trifenatate; T1, baseline; T2, Day 15; T3, Day 21

**Fig. 3:**
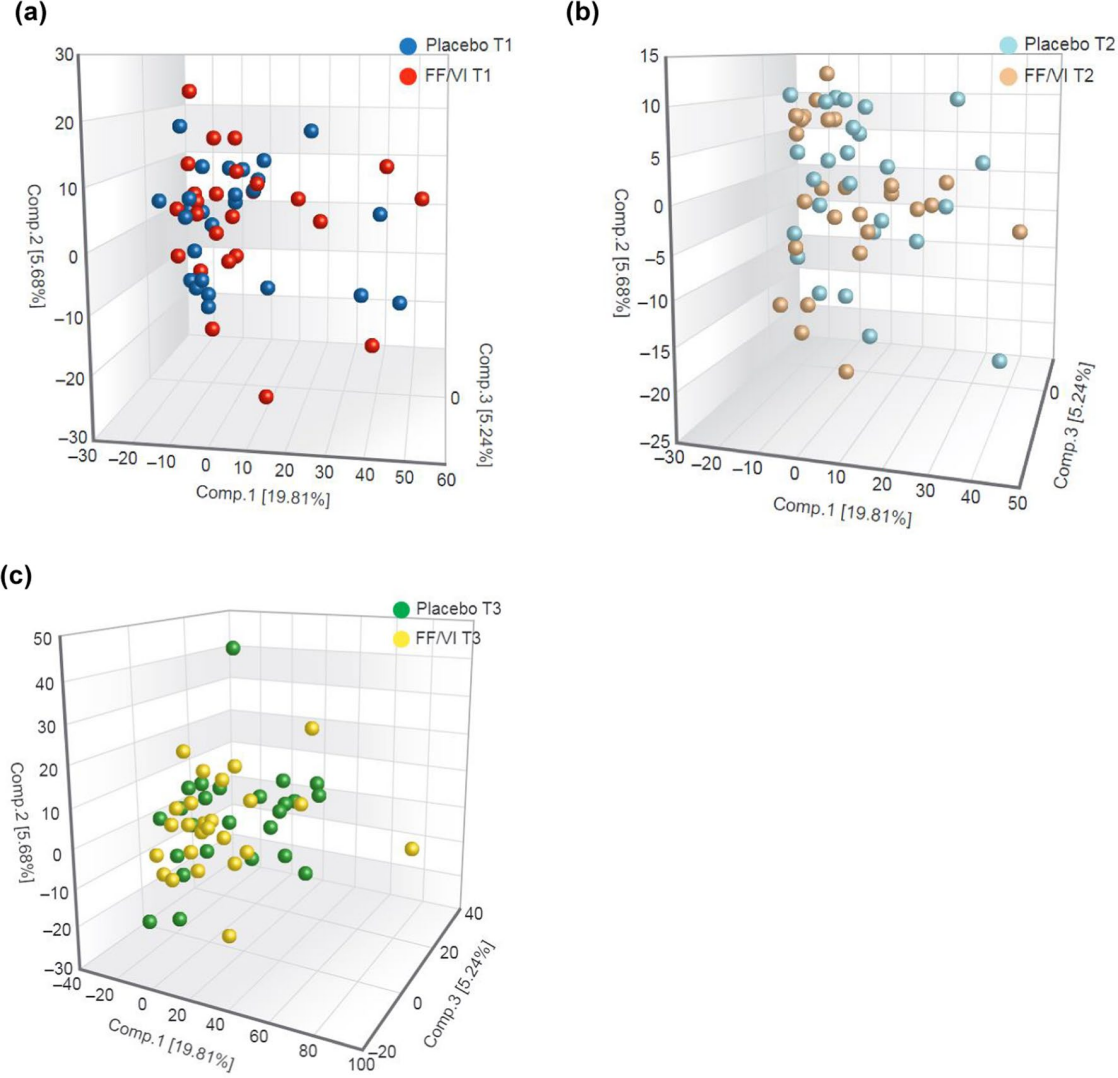
PCA plot for **a** T1, **b** T2 and **c** T3. Three-dimensional PCA plot displaying each individual serum sample from each timepoint (placebo and FF/VI), accounting for all metabolites. Comp., component; FF/VI, fluticasone furoate/vilanterol trifenatate; PCA, principal component analysis; T1, baseline; T2, Day 15; T3, Day 21

PCA plot analysis reported no visible high-level separation between placebo- and FF/VI-treated groups at baseline (T1, Fig. 3a), very little high-level separation between placebo and FF/VI treatments at T2 (Fig. 3b) and modest high-level separation between treatment and placebo samples at T3 (Fig. 3c).

At T1, RF analyses suggested that there were no differences between the overall metabolic profiles of the FF/VI and placebo-treated groups, as shown by an error rate of 51.92%, indicating that the two groups could be separated metabolically with a success rate of 48.08%, as compared with 50.00% by random chance alone. At T2, changes were small, with the RF analysis indicating an improved success rate of 65.38%, which is in line with the rise in the number of individual significant metabolite perturbations at T2, as compared with T1. At T3, the RF analysis was similar to that of T2, with a success rate of 59.62%.

**Fig. 4:**
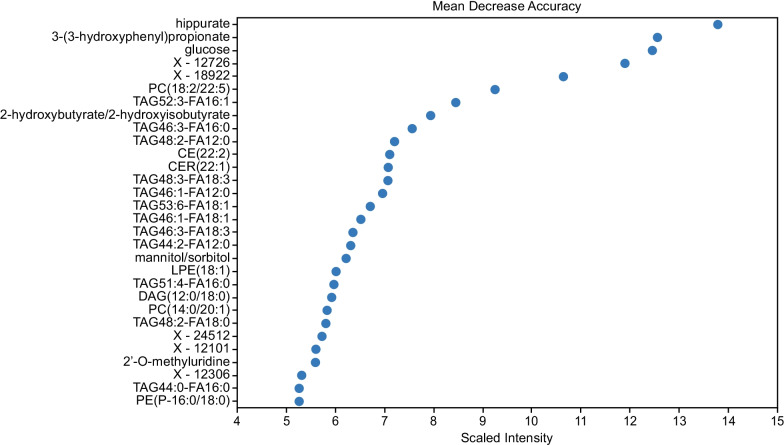
RF importance plot comparing FF/VI with placebo at T3. Metabolites listed at the left of the graph are ordered by importance in their role to separate treatment groups (FF/VI vs placebo). The x-axis shows the “Mean Decrease Accuracy,” which is a measure of the importance of a particular biochemical to the accurate classification of a given sample in the appropriate group. FF/VI, fluticasone furoate/vilanterol trifenatate; RF, random forest; T3, Day 21

For the T3 comparison, an RF importance plot highlighted the specific biochemicals contributing to the minor diversity between FF/VI and placebo (Fig. 4). Metabolites were identified and ranked by their importance in separating serum samples from the FF/VI and placebo treatment groups. The most important metabolites are more critical for the separation of the groups as they drove the statistical ability of the tool to accurately classify serum from participants given either placebo or FF/VI. Of note, two bacterially derived benzoate metabolites (hippurate and 3-[3-hydroxyphenyl] propionate) were the most important for the separation of the treatment groups, with the majority of the top 30 important biochemicals being dominated by complex lipid species. Quality control statistics suggested that all data produced by the metabolomic platform met process specifications (Additional file 1: Table S1).

### Associations between airway inflammation and systemic metabolomics

As previously reported, treatment with FF/VI reduced baseline FeNO levels (Fig. 1) [6]. However, in this secondary analysis, systemic intermediates involved in nitric oxide (NO) biosynthesis yielded no statistically significant changes, suggesting minimal systemic impact of FF/VI within this node of metabolism. There were no significant statistical correlations between any arginine metabolism-related biochemicals and exhaled NO levels. The most notable and robust correlation analysis signatures were found within the metabolic nodes of acylcarnitines and androgenic steroids and baseline exhaled NO levels. These differences may have been driven by the participants’ sex. Negative correlations between acylcarnitine and exhaled NO levels were yielded only by female participants, whilst correlations between androgenic steroids and exhaled NO levels were largely driven by male participants (Table 3).

**Table 3:** Statistical summary of biochemicals related to fatty acid β-oxidation and androgen metabolism

Biochemical name	Repeated measures ANOVA			Pearsons correlations (to baseline FeNO)		
	T1 FF/VI vs placebo	T2 FF/VI vs placebo	T3 FF/VI vs placebo	All participants	Females	Males
*Fatty acid metabolism (acyl carnitine)*						
Acetylcarnitine (C2)	1 p = 0.876 q = 1	0.98 p = 0.411 q = 0.608	0.91 p = 0.204 q = 0.462	− 0.1377 p = 0.163 q = 0.577	− 0.4939 p = 0.001 q = 0.08	0.0648 p = 0.611 q = 0.999
Hexanoylcarnitine (C6)	1 p = 0.984 q = 1	1.27 p = 0.517 q = 0.629	1.33 p = 0.112 q = 0.375	− 0.2619 p = 0.007 q = 0.227	− 0.4849 p = 0.002 q = 0.096	− 0.0048 p = 0.97 q = 0.999
Octanoylcarnitine (C8)	0.97 p = 0.831 q = 1	1.44 p = 0.532 q = 0.633	1.28 p = 0.340 q = 0.586	− 0.2086 p = 0.034 q = 0.367	− 0.4515 p = 0.004 q = 0.128	0.0438 p = 0.731 q = 0.999
Decanoylcarnitine (C10)	1.03 p = 0.885 q = 1	1.37 p = 0.663 q = 0.665	1.34 p = 0.378 q = 0.611	− 0.2389 p = 0.015 q = 0.264	− 0.4661 p = 0.002 q = 0.112	0.0109 p = 0.932 q = 0.999
5-Dodecenoylcarnitine (C12:1)	0.93 p = 0.634 q = 1	1.16 p = 0.9 q = 0.729	1.25 p = 0.193 q = 0.450	− 0.2437 p = 0.013 q = 0.258	− 0.443 p = 0.004 q = 0.138	− 0.0447 p = 0.726 q = 0.999
*Cis*-4-decenoylcarnitine (C10:1)	1.05 p = 0.858 q = 1	1.19 p = 0.732 q = 0.683	1.28 p = 0.221 q = 0.482	− 0.2956 p = 0.002 q = 0.152	− 0.4993 p = 0.001 q = 0.077	− 0.1001 p = 0.431 q = 0.999
Laurylcarnitine (C12)	1 p = 0.901 q = 1	1.21 p = 0.828 q = 0.713	1.11 p = 0.44 q = 0.645	− 0.2088 p = 0.033 q = 0.367	− 0.4183 p = 0.007 q = 0.172	− 0.0021 p = 0.987 q = 0.999
Myristoylcarnitine (C14)	0.93 p = 0.376 q = 1	1.04 p = 0.965 q = 0.745	1.05 p = 0.646 q = 0.721	− 0.1784 p = 0.07 q = 0.458	− 0.4132 p = 0.008 q = 0.172	− 0.0096 p = 0.94 q = 0.999
Palmitoylcarnitine (C16)	0.9 p = 0.104 q = 1	0.96 p = 0.698 q = 0.672	1.06 p = 0.373 q = 0.609	− 0.0532 p = 0.592 q = 0.764	− 0.337 p = 0.033 q = 0.282	0.1153 p = 0.364 q = 0.999
Palmitoleoylcarnitine (C16:1)	0.94 p = 0.411 q = 1	1.04 p = 0.845 q = 0.719	1.14 p = 0.163 q = 0.418	− 0.1618 p = 0.101 q = 0.513	− 0.4426 p = 0.004 q = 0.138	0.0571 p = 0.654 q = 0.999
Stearoylcarnitine (C18)	0.92 p = 0.142 q = 1	1 p = 0.97 q = 0.747	1.04 p = 0.483 q = 0.673	− 0.0326 p = 0.742 q = 0.805	− 0.0742 p = 0.649 q = 0.738	0.014 p = 0.912 q = 0.999
Linoleoylcarnitine (C18:2)	0.95 p = 0.22 q = 1	1 p = 0.865 q = 0.725	1.03 p = 0.699 q = 0.742	− 0.141 p = 0.153 q = 0.561	− 0.3014 p = 0.059 q = 0.357	− 0.0324 p = 0.799 q = 0.999
Linolenoylcarnitine (C18:3)	1.07 p = 0.956 q = 1	0.97 p = 0.79 q = 0.7	0.93 p = 0.85 q = 0.781	− 0.1397 p = 0.157 q = 0.568	− 0.2658 p = 0.097 q = 0.402	− 0.0294 p = 0.818 q = 0.999
Oleoylcarnitine (C18:1)	0.94 p = 0.314 q = 1	0.99 p = 0.71 q = 0.675	1.03 p = 0.509 q = 0.68	− 0.0856 p = 0.388 q = 0.699	− 0.1733 p = 0.285 q = 0.567	0.0219 p = 0.864 q = 0.999
Myristoleoylcarnitine (C14:1)	0.99 p = 0.673 q = 1	1.21 p = 0.798 q = 0.702	1.2 p = 0.228 q = 0.488	− 0.1977 p = 0.044 q = 0.395	− 0.3947 p = 0.012 q = 0.203	0.0086 p = 0.947 q = 0.999
Adipoylcarnitine (C6-DC)	1.1 p = 0.517 q = 1	1 p = 0.905 q = 0.729	1.12 p = 0.256 q = 0.519	− 0.0321 p = 0.746 q = 0.806	− 0.3746 p = 0.017 q = 0.233	0.1402 p = 0.269 q = 0.999
Octadecanedioylcarnitine (C18-DC)	1.03 p = 0.742 q = 1	1.05 p = 0.993 q = 0.748	1.16 p = 0.406 q = 0.632	0.0931 p = 0.347 q = 0.684	− 0.0138 p = 0.932 q = 0.804	0.138 p = 0.277 q = 0.999
Octadecenedioylcarnitine (C18:1-DC)	1.03 p = 0.85 q = 1	1.05 p = 0.859 q = 0.724	1.45 p = 0.056 q = 0.339	− 0.0003 p = 0.998 q = 0.845	− 0.1592 p = 0.327 q = 0.589	0.0433 p = 0.734 q = 0.999
Arachidoylcarnitine (C20)	0.93 p = 0.263 q = 1	1.14 p = 0.253 q = 0.579	1.01 p = 0.773 q = 0.762	0.0771 p = 0.436 q = 0.717	− 0.0044 p = 0.978 q = 0.814	0.1588 p = 0.21 q = 0.999
Arachidonoylcarnitine (C20:4)	0.95 p = 0.587 q = 1	1.02 p = 0.781 q = 0.698	1 p = 0.996 q = 0.805	0.0524 p = 0.597 q = 0.764	0.0461 p = 0.778 q = 0.771	0.0656 p = 0.606 q = 0.999
Behenoylcarnitine (C22)	1.19 p = 0.088 q = 1	1.1 p = 0.52 q = 0.629	1.13 p = 0.131 q = 0.391	− 0.043 p = 0.664 q = 0.792	0.0041 p = 0.98 q = 0.814	− 0.0477 p = 0.708 q = 0.999
Dihomo-linolenoylcarnitine (C20:3n3 or 6)	0.94 p = 0.352 q = 1	0.99 p = 0.833 q = 0.714	0.97 p = 0.587 q = 0.702	0.0565 p = 0.569 q = 0.764	0.1763 p = 0.276 q = 0.566	0.0249 p = 0.845 q = 0.999
Dihomo-linoleoylcarnitine (C20:2)	0.99 p = 0.891 q = 1	0.98 p = 0.467 q = 0.622	1 p = 0.828 q = 0.779	− 0.0242 p = 0.808 q = 0.813	− 0.0841 p = 0.606 q = 0.73	0.0695 p = 0.585 q = 0.999
Eicosenoylcarnitine (C20:1)	1.01 p = 0.882 q = 1	1 p = 0.635 q = 0.657	1.12 p = 0.266 q = 0.526	0.0162 p = 0.87 q = 0.826	− 0.0763 p = 0.64 q = 0.736	0.1067 p = 0.402 q = 0.999
Docosapentaenoylcarnitine (C22:5n3)	0.79 p = 0.016 q = 1	0.9 p = 0.335 q = 0.584	0.87 p = 0.338 q = 0.585	0.1399 p = 0.157 q = 0.568	0.1823 p = 0.26 q = 0.556	0.1178 p = 0.354 q = 0.999
Lignoceroylcarnitine (C24)	1.04 p = 0.865 q = 1	1.07 p = 0.571 q = 0.648	1.04 p = 0.566 q = 0.695	0.0719 p = 0.468 q = 0.727	− 0.0944 p = 0.562 q = 0.713	0.1375 p = 0.279 q = 0.999
Margaroylcarnitine (C17)	0.94 p = 0.584 q = 1	0.92 p = 0.443 q = 0.614	1.12 p = 0.425 q = 0.641	− 0.026 p = 0.794 q = 0.813	− 0.08 p = 0.624 q = 0.733	0.0388 p = 0.761 q = 0.999
Nervonoylcarnitine (C24:1)	1.01 p = 0.65 q = 1	0.91 p = 0.123 q = 0.541	1.16 p = 0.184 q = 0.445	0.0577 p = 0.561 q = 0.764	0.0441 p = 0.787 q = 0.774	0.0467 p = 0.714 q = 0.999
Cerotoylcarnitine (C26)	0.94 p = 0.119 q = 1	0.99 p = 0.896 q = 0.729	1.06 p = 0.998 q = 0.805	0.1332 p = 0.178 q = 0.585	0.0706 p = 0.665 q = 0.741	0.1323 p = 0.298 q = 0.999
Ximenoylcarnitine (C26:1)	0.98 p = 0.512 q = 1	0.95 p = 0.326 q = 0.579	1.05 p = 0.934 q = 0.791	0.1639 p = 0.097 q = 0.513	0.1276 p = 0.433 q = 0.649	0.1268 p = 0.318 q = 0.999
*Androgenic steroids*						
11-Ketoetiocholanolone glucuronide	0.75 p = 0.136 q = 1	0.85 p = 0.328 q = 0.579	0.92 p = 0.622 q = 0.71	− 0.1919 p = 0.051 q = 0.421	− 0.2557 p = 0.111 q = 0.42	− 0.1005 p = 0.429 q = 0.999
Dehydroisoandrosterone sulfate (DHEA-S)	0.95 p = 0.234 q = 1	0.96 p = 0.274 q = 0.579	0.96 p = 0.388 q = 0.617	− 0.1328 p = 0.179 q = 0.587	0.0812 p = 0.619 q = 0.733	− 0.2205 p = 0.08 q = 0.857
16alpha-Hydroxy DHEA 3-sulfate	1.03 p = 0.999 q = 1	0.90 p = 0.462 q = 0.622	1.12 p = 0.086 q = 0.347	− 0.0825 p = 0.405 q = 0.703	0.0506 p = 0.757 q = 0.763	− 0.0532 p = 0.676 q = 0.999
Androsterone glucuronide	0.95 p = 0.677 q = 1	0.95 p = 0.414 q = 0.608	1 p = 0.874 q = 0.786	− 0.2311 p = 0.018 q = 0.285	− 0.0477 p = 0.77 q = 0.767	− 0.2862 p = 0.022 q = 0.757
Epiandrosterone sulfate	1.01 p = 0.778 q = 1	1 p = 0.842 q = 0.719	1.01 p = 0.747 q = 0.752	− 0.149 p = 0.131 q = 0.546	0.0701 p = 0.667 q = 0.742	− 0.246 p = 0.05 q = 0.795
Androsterone sulfate	0.99 p = 0.949 q = 1	0.97 p = 0.866 q = 0.726	1.07 p = 0.601 q = 0.705	− 0.2195 p = 0.025 q = 0.328	− 0.1197 p = 0.462 q = 0.671	− 0.2444 p = 0.052 q = 0.795
Etiocholanolone glucuronide	0.89 p = 0.197 q = 1	0.90 p = 0.168 q = 0.568	0.97 p = 0.623 q = 0.71	− 0.1111 p = 0.262 q = 0.652	− 0.0016 p = 0.992 q = 0.815	− 0.1139 p = 0.37 q = 0.999
5alpha-Androstan-3alpha,17alpha-diol monosulfate	0.96 p = 0.954 q = 1	0.84 p = 0.09 q = 0.541	1.02 p = 0.837 q = 0.781	− 0.2525 p = 0.01 q = 0.249	− 0.1143 p = 0.483 q = 0.676	− 0.3115 p = 0.012 q = 0.757
Androstene-3beta,17beta-diol monosulfate (1)	0.96 p = 0.344 q = 1	0.98 p = 0.877 q = 0.727	0.77 p = 0.188 q = 0.446	0.0214 p = 0.829 q = 0.818	0.0915 p = 0.574 q = 0.717	− 0.0004 p = 0.997 q = 1
Androstene-3beta,17beta-diol monosulfate (2)	1 p = 0.547 q = 1	0.98 p = 0.586 q = 0.652	0.93 p = 0.165 q = 0.42	− 0.1562 p = 0.113 q = 0.521	0.1053 p = 0.518 q = 0.693	− 0.2576 p = 0.04 q = 0.757
Androstene-3beta,17beta-diol disulfate (1)	0.95 p = 0.116 q = 1	0.99 p = 0.409 q = 0.608	0.93 p = 0.086 q = 0.347	− 0.0733 p = 0.46 q = 0.726	0.0532 p = 0.745 q = 0.762	− 0.0828 p = 0.516 q = 0.999
Androstene-3beta,17beta-diol disulfate (2)	0.97 p = 0.72 q = 1	0.95 p = 0.101 q = 0.541	0.96 p = 0.375 q = 0.609	− 0.2215 p = 0.024 q = 0.323	− 0.211 p = 0.191 q = 0.501	− 0.2386 p = 0.058 q = 0.823
Androstene-3alpha,17alpha-diol monosulfate (2)	0.99 p = 0.237 q = 1	1 p = 0.995 q = 0.748	0.93 p = 0.995 q = 0.805	− 0.1628 p = 0.099 q = 0.513	− 0.2825 p = 0.077 q = 0.378	− 0.0132 p = 0.918 q = 0.999
Androstene-3alpha,17alpha-diol monosulfate (3)	0.92 p = 0.205 q = 1	0.92 p = 0.181 q = 0.573	1.03 p = 0.659 q = 0.725	− 0.2438 p = 0.013 q = 0.258	− 0.2186 p = 0.175 q = 0.487	− 0.2559 p = 0.041 q = 0.761
5alpha-Androstan-3alpha,17beta-diol monosulfate (1)	0.99 p = 0.764 q = 1	1 p = 0.798 q = 0.702	0.78 p = 0.338 q = 0.585	0.0101 p = 0.919 q = 0.829	0.0504 p = 0.757 q = 0.763	0.0228 p = 0.858 q = 0.999
5alpha-Androstan-3alpha,17beta-diol monosulfate (2)	1.02 p = 0.694 q = 1	0.93 p = 0.266 q = 0.579	1 p = 0.588 q = 0.702	− 0.2519 p = 0.01 q = 0.249	− 0.2998 p = 0.06 q = 0.357	− 0.2942 p = 0.018 q = 0.757
5alpha-Androstan-3alpha,17beta-diol disulfate	0.96 p = 0.717 q = 1	0.91 p = 0.119 q = 0.541	0.95 p = 0.817 q = 0.774	− 0.1641 p = 0.096 q = 0.513	− 0.2164 p = 0.18 q = 0.493	− 0.1234 p = 0.331 q = 0.999
5alpha-Androstan-3alpha,17beta-diol 17-glucuronide	1.11 p = 0.316 q = 1	0.99 p = 0.807 q = 0.704	0.8 p = 0.505 q = 0.68	0.0211 p = 0.832 q = 0.818	0.2534 p = 0.115 q = 0.422	− 0.0797 p = 0.532 q = 0.999
5alpha-Androstan-3beta,17beta-diol monosulfate (2)	1.04 p = 0.574 q = 1	1 p = 0.862 q = 0.724	0.76 p = 0.239 q = 0.499	0.0292 p = 0.769 q = 0.806	0.125 p = 0.442 q = 0.656	0.0059 p = 0.963 q = 0.999
5alpha-Androstan-3beta,17beta-diol disulfate	0.99 p = 0.605 q = 1	1.03 p = 0.645 q = 0.66	1.01 p = 0.216 q = 0.477	− 0.0349 p = 0.725 q = 0.8	0.1332 p = 0.412 q = 0.636	–0.0985 p = 0.439 q = 0.999
5alpha-Androstan-3beta,17alpha-diol disulfate	1.02 p = 0.268 q = 1	1.05 p = 0.754 q = 0.693	0.94 p = 0.602 q = 0.705	− 0.1619 p = 0.101 q = 0.513	− 0.1672 p = 0.302 q = 0.579	− 0.1301 p = 0.305 q = 0.999
Andro steroid monosulfate C_19_H_28_O_6_S (1)	0.99 p = 0.915 q = 1	0.95 p = 0.457 q = 0.622	1.08 p = 0.196 q = 0.455	− 0.0956 p = 0.335 q = 0.679	0.0734 p = 0.653 q = 0.738	–0.0975 p = 0.444 q = 0.999

ANOVA, analysis of variance; FeNO, fraction of exhaled nitric oxide; FF/VI, fluticasone furoate/vilanterol trifenatate; T1, baseline; T2, Day 15; T3, Day 21

### Systemic anti-inflammatory activity

**Fig. 5:**
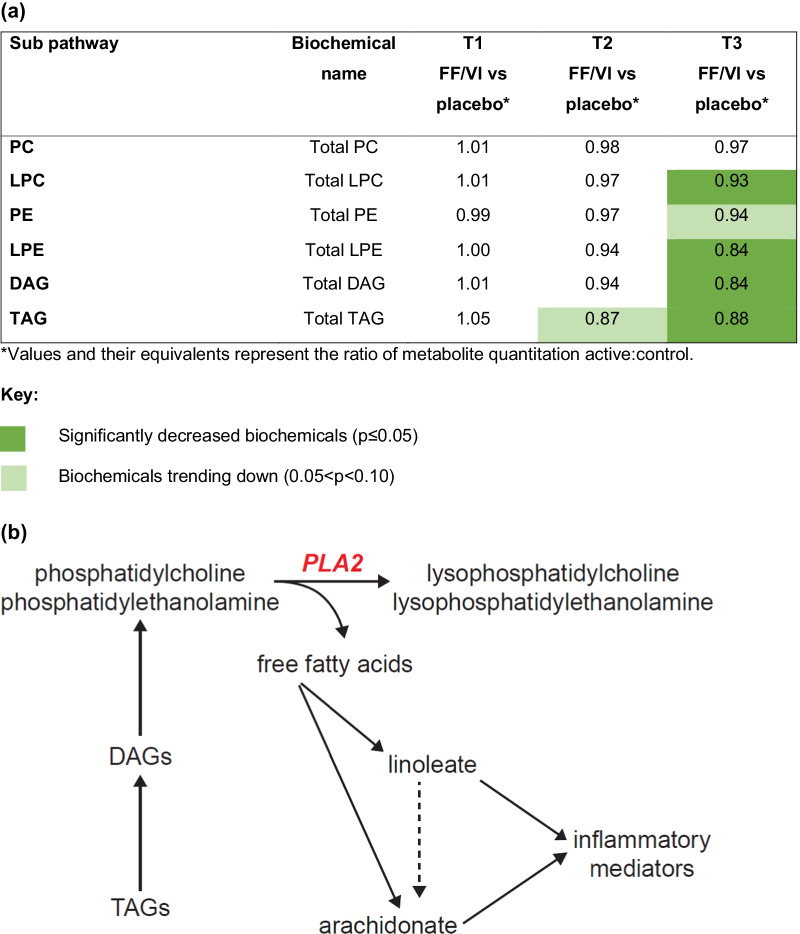
**a** Heat map and **b** biochemical pathway for complex lipids. DAG, diacylglycerol; FF/VI, fluticasone furoate/vilanterol trifenatate; LPC, lysophosphatidylcholine; LPE, lysophosphatidylethanolamine; PC, phosphatidylcholine; PE, phosphatidylethanolamine; PLA2, phospholipase A2; TAG, triacylglycerol; T1, baseline; T2, Day 15; T3, Day 21

Systemic anti-inflammatory activity was evident within complex lipid metabolism, including a reduction in lysophosphatidylcholines (LPC) and lysophosphatidylethanolamines (LPE) (Fig. 5a, heat map). Both LPC and LPE are metabolites of phospholipase A2 (PLA2) (Fig. 5b, biochemical pathway), with a reduction suggesting reduced PLA2 activity. However, this suggestion cannot be strengthened by evidence from other metabolite changes as there were no significant changes in downstream free fatty acids or lipid inflammatory mediators such as eicosanoids.

**Fig. 6:**
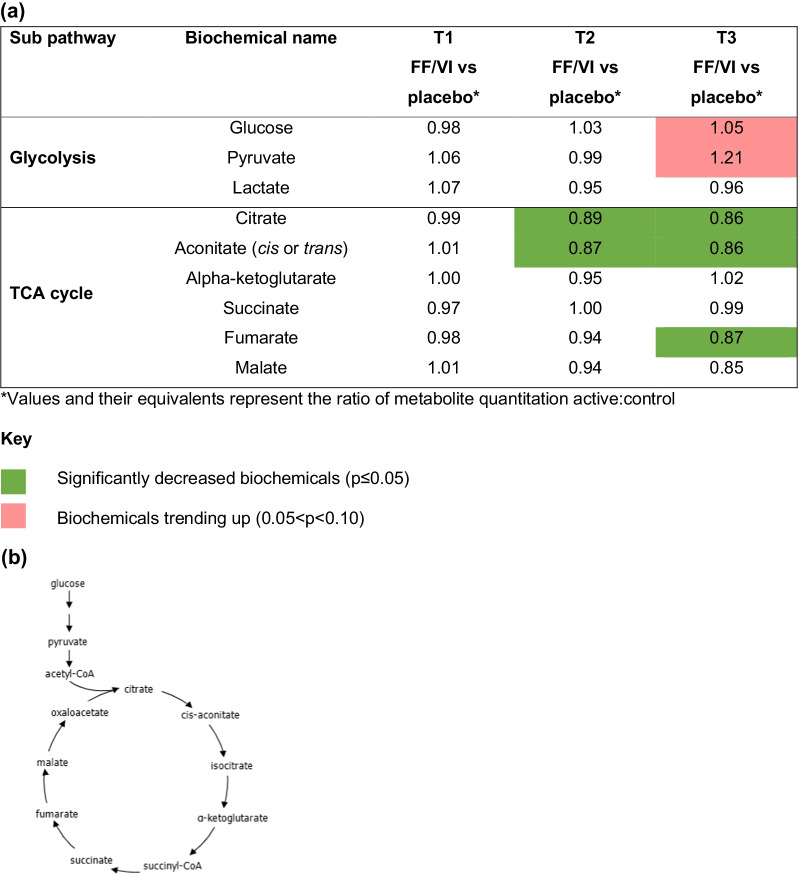
**a** Heat map and **b** biochemical pathway for fatty acids. Acetyl-CoA, acetyl coenzyme A; FF/VI, fluticasone furoate/vilanterol trifenatate; succinyl-CoA, succinyl coenzyme A; TCA, tricarboxylic acid; T1, baseline; T2, Day 15; T3, Day 21

**Fig. 7:**
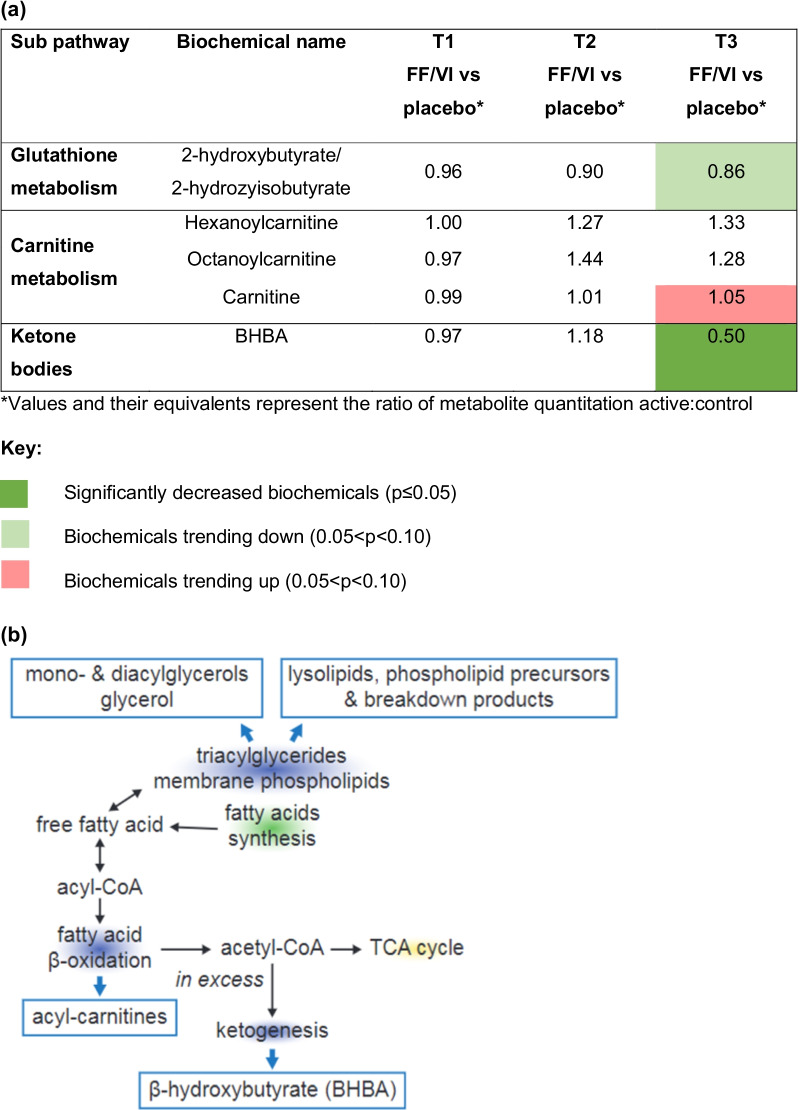
**a** Heat map and **b** biochemical pathway for antioxidants. Acetyl-CoA, acetyl coenzyme A; acyl-CoA, acyl coenzyme A; BHBA, β-hydroxybutyrate; FF/VI, fluticasone furoate/vilanterol trifenatate; TCA, tricarboxylic acid; T1, baseline; T2, Day 15; T3, Day 21

**Fig. 8:**
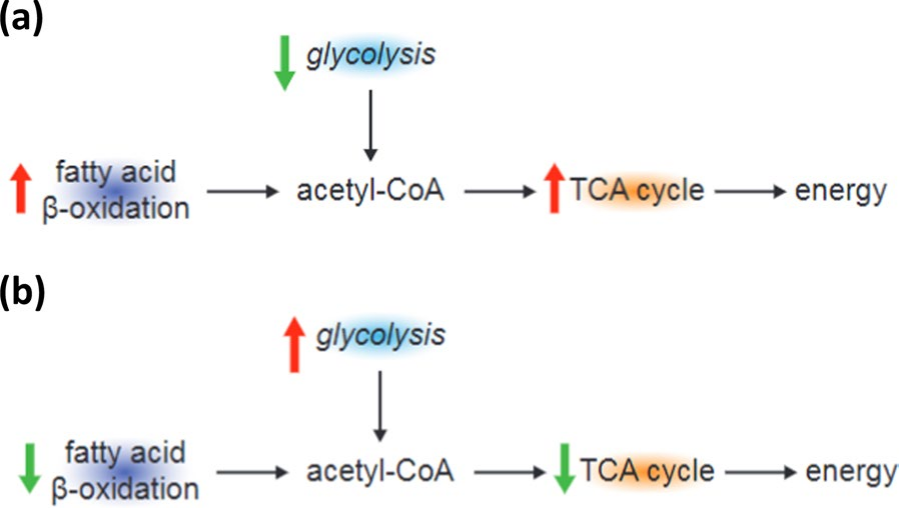
Energetics profiles for both (**a**) patients with asthma (from the literature) and (**b**) participants treated with active drug versus placebo in this study. Acetyl-CoA, acetyl coenzyme A; TCA, tricarboxylic acid

At T3, there was biochemical evidence of increased glycolysis with elevated pyruvate levels and less efficient oxidative phosphorylation activity with reduced levels of the tricarboxylic acid cycle (TCA) intermediates citrate, aconitate and fumarate compared with placebo (Fig. 6). Citrate and aconitate were also significantly reduced at T2 compared with placebo. There was evidence for increased lipid metabolism in the placebo group compared with FF/VI, as suggested by elevated production of 3-hydroxybutyrate via ketogenesis (Fig. 7). This may be related to the observation of a potential increase in lipid metabolism via PLA2 activity, which would increase fatty acid mobilization and availability for fatty acid β-oxidation (Fig. 8). In the active treatment arm, this pattern was reversed.

**Fig. 9:**
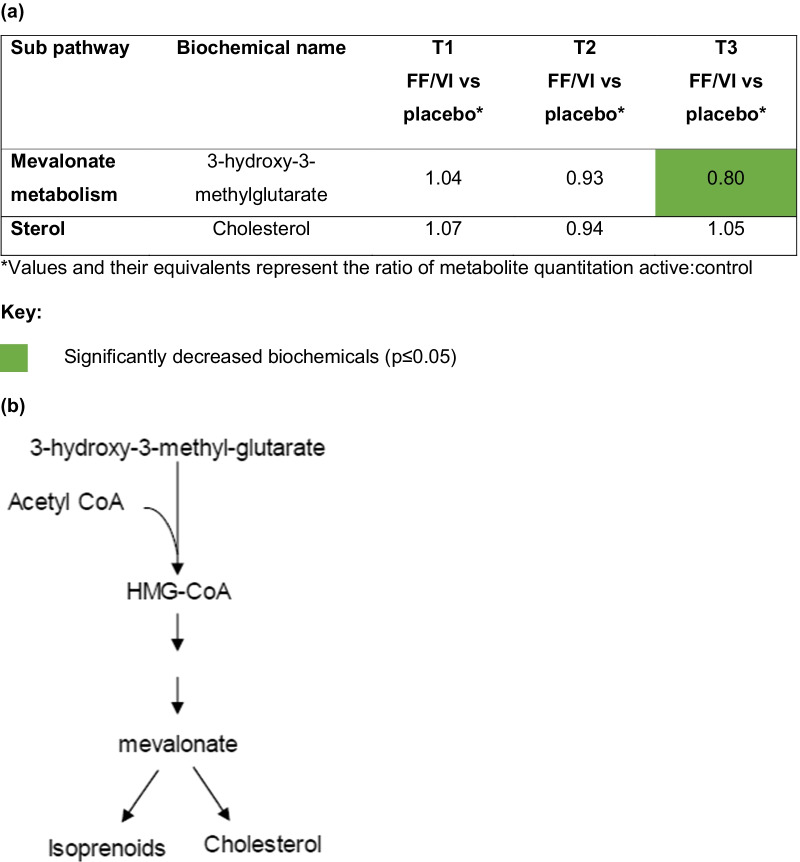
**a** Heat map and **b** biochemical pathway for mevalonate biosynthesis and the generation of isoprenoids and cholesterol. Acetyl-CoA, acetyl coenzyme A; FF/VI, fluticasone furoate/vilanterol trifenatate; HMG-CoA, 3-hydroxy-3-methyl-glutarate coenzyme A; T1, baseline; T2, Day 15; T3, Day 21

Treatment with FF/VI was also associated with a reduction in the mevalonate precursor 3-hydroxy-3-methyl-glutarate, which could potentially suggest an initiation of inflammatory mechanisms associated with blockage of the mevalonate pathway. However, this was not associated with elevated cholesterol and lipoprotein levels (Fig. 9).

### Systemic bioactivity on endocrine, metabolic or other pathways of glucocorticoid action

There was no evidence for potentially adverse systemic bioactivity, including systemic effects on endogenous steroids or endocrine systems (e.g. cortisol, cortisone dehydroepiandrosterone sulfate or corticosterone). For example, although cortisol is highly responsive to exogenous glucocorticoid exposure, the changes (ratio to placebo) seen (0.9 and 1.02 at T2 and T3, respectively) were small and not statistically significant. Moreover, markers of stress response (e.g. homovanillic acid, 3,4-hydroxyphenyl glycerol or 3,4-hydroxyphenyl acetate), insulin resistance (e.g. *N*-methyl nicotinamide), muscle protein turnover (e.g. 3-methyl histidine, histidine, tryptophan, glycine, arginine, threonine, tyrosine or glutamine) and bone metabolism (e.g. prolyl hydroxyproline, pipecolate, *N*-palmityl taurine or *trans* 4-hydroxy proline) did not yield significant or detectable changes (see Additional file 2 for full metabolomic analysis).

### Safety

As previously reported, 17 participants in the primary analysis population (63%; N = 27, includes one participant who did not provide serum samples) reported adverse events (AEs) during the study. Only one AE, cough with moderate intensity which resolved prior to cessation of study medication, was reported as potentially drug related. There were no serious AEs, and no deaths [6].

## Discussion

This study investigated the global metabolomic and lipidomic changes following administration of FF/VI or placebo. Although this was not a priori targeted analysis, in the interpretation of the results, there was a focus on the metabolic pathways known to be associated with asthma pathogenesis, particularly those potentially related to airway inflammation, and on metabolic pathways known to be associated with corticosteroid systemic activity. Based on findings in other studies [10], it was expected that FF/VI would exhibit low levels of systemic bioactivity since both components are inhaled only once daily at low doses [4], have prolonged lung retention, low oral bioavailability and high systemic clearance [3, 6]. The global untargeted metabolomic and complex lipid analysis of serum from adults with asthma revealed subtle, but potentially important, biochemical differences between participants who received placebo and those who received FF/VI. This is in contrast to the pronounced effects in the airways assessed via exhaled nitric oxide, which suggests that the treatment effect was largely located within the airways [6]. The largest number of significant changes were yielded when comparing FF/VI with placebo, suggesting that some drug-dependent effects may persist systemically following treatment and into the washout phase of the study. Overall, the magnitude of changes was small compared with results obtained in studies of other ICS/LABA combinations. For example, FF 100 µg has been previously shown to produce a 7% reduction in plasma cortisol, which although not statistically significant, is a dose-related effect [11]. Similarly, in a large metabolic profiling study by Kachroo et al. [12]*,* a dose–effect relationship was observed with steroid metabolites including cortisol and dehydroisoandrosterone sulfate and ICS treatment. Although this reduction was primarily driven by ICS treatment, reduced steroid levels also represented a fundamental characteristic of pathophysiology of asthma [12]. It is important to note that cortisol suppression is not the same as adrenal suppression; nevertheless, reductions in steroid metabolites have been referred to as “adrenal insufficiency” by some authors [12], but this was without evidence via adrenocorticotropic hormone stimulation testing. Since the magnitude of the steroid metabolite reductions following low-dose ICS are generally small and hence readily reversible on discontinuation of ICS therapy, they may not be clinically significant in the majority of cases. Indeed, adrenal insufficiency is classified as a very rare (< 1/10,000) [13] to rare (≥ 1/10,000 to < 1/1000) [14] adverse effect of ICS therapy. In this study, there was a 10% reduction in cortisol at T2 and a 2% increase in cortisol at T3, both of which were not statistically significant. These findings align with those recently reported by Daley-Yates et al. [15] in a comparative metabolomic study of inhaled FF, fluticasone propionate and budesonide across a wide range of doses. The most sensitive and dose-responsive biomarkers of exogenous glucocorticoid systemic exposure were the glucuronide metabolites of cortisol and cortisone, and the pregnenolone metabolite dehydroepiandrosterone sulfate. In the therapeutic dose range, FF 100 µg/day had the least effect on these biomarkers. Across the entire therapeutic dose ranges of the three ICS molecules, FF produced mean changes in a smaller percentage of the quantifiable metabolites (0.96% [0.36%↑/0.61%↓]) compared with equivalent therapeutic doses of the other ICS (fluticasone propionate 500 µg/day [1.67% (0.46%↑/1.22%↓)] and budesonide 800 µg/day [1.42% (0.56%↑/0.91%↓)]) [15].

In this study, we show that the long duration of topical anti-inflammatory action of FF, based on FeNO suppression (Fig. 1), as previously described by Bardsley et al., was associated with systemic anti-inflammatory signals that were of a low magnitude compared with topical effect in the airways as indicated by a 60% reduction in FeNO in these participants [6]. Despite this, correlations were seen between acylcarnitines and androgenic steroids, and airway inflammation as assessed by baseline FeNO levels [6]. This may be reflective of the role of androgens in both asthma and inflammation pathogenesis, although FF/VI by itself did not perturb androgen levels [16]. In contrast, Kachroo et al. [12] observed significant reductions in androgen, corticosteroid and pregnenolone metabolites in ICS-treated asthma patients who received predominantly budesonide, an ICS shown to have greater systemic activity at therapeutically equivalent doses to FF [11]. They also noted that part of the steroid metabolite reduction was related to asthma pathophysiology.

The small magnitude of changes seen in the global metabolome in this study provide evidence that significant systemic bioactivity on endocrine, metabolic or other pathways of glucocorticoid action are unlikely to occur after 14 days of treatment with FF/VI. There was also a lack of significant or detectable changes in markers of stress response, insulin resistance, muscle protein turnover and bone metabolism within the global metabolome. Both findings contrast with previous studies where systemic corticosteroids yielded extensive effects on the metabolome [10] or ICS at higher and supratherapeutic doses had a marked effect on multiple metabolites [15], including two biomarkers of adrenal suppression: dehydroisoandrosterone sulfate and cortisol, that could be used to monitor for potential AEs while maintaining the benefits of ICS treatment for patients with asthma [12].

A reduction in LPC and LPE following treatment with FF/VI suggests reduced PLA2 activity. PLA2 activity has been recognized to play a role in the pathophysiology of asthma development, with elevated activity reported in participants with acute asthma [17]. Therapy with FF/VI elicits altered complex lipid metabolism which potentially impacts PLA2 activity. However, there was no detectable effect on downstream free fatty acids or lipid inflammatory mediators. The lipid changes and an association with PLA2 in this metabolomic analysis is plausible given that anti-inflammatory glucocorticoids have been primarily shown to inhibit the transcription of cytosolic PLA2 and inhibit cyclo-oxygenase-2 gene expression, leading to reduced eicosanoid production [17].

Treatment with FF/VI provokes differential energetics profiles within the nodes of β-oxidation, glycolysis and the TCA cycle, with a higher lipid metabolism and β-oxidation in those treated with placebo, which is consistent with asthma pathogenesis [16]. This pattern was reversed in those receiving FF/VI.

The gut microbiota can play a role in multiple metabolic conditions, with alterations inducing both pro- and anti-inflammatory effects [18]. A preclinical study has demonstrated that chronic exposure to glucocorticoids can lead to both positive and negative shifts in the gut microbiota [18]. The metabolomic assessment of FF/VI demonstrated a potential treatment effect on the host microbiome 7 days after treatment cessation (T3), with a reduction in hippurate and a few related biochemicals associated with microbial degradation of certain dietary components. As this effect was evident only after treatment cessation, the relevance is unknown. However, exposure of gut bacteria to FF/VI is likely to occur since some dose is swallowed during inhalation. As study participants were admitted to an inpatient research unit from day 14 to day 19 it is also possible that dietary changes may have contributed to this effect.

Excessive NO generation in asthma can result in cell damage in the airways due to a long-term deleterious effect and possible involvement in eosinophilic inflammation [19, 20]. As previously reported for the primary analysis, treatment with FF/VI for 14 days was associated with a 60% reduction in FeNO, compared with placebo, indicating a pronounced anti-inflammatory action [6]. In this secondary, metabolomic analysis, there were only subtle systemic effects on NO metabolism observed with treatment. This suggests a localization of reduced NO generation in the airways.

A potential limitation of this study is that the dosing period was only 14 days and therefore may not be generalizable to long-term use, which may explain some of the differences in our findings compared with the longer-term metabolic study carried out by Kachroo et al. [12], although other studies have shown that even a single dose of glucocorticoids can perturb numerous metabolic pathways [10]. The sample size of 26 adults with asthma and extent of demographic diversity was also relatively limited, with a greater proportion of men than women and no participants with severe asthma. The exploratory nature of this analysis coupled with estimated false discovery rates of 32% and above reported in this study mean that future long-term or additional short-term studies could provide additional strength to our results. It should be noted that quality control relative standard deviations were higher for the complex lipid panel, which increases the uncertainty as to the significance of findings for this panel. Although quality control data indicated that data from the metabolomic platform met process specifications, it is possible that differences between samples (median relative standard deviation [RSD] 13%) may have been dominated by complex lipid species due to variability within the assay, as opposed to treatment-related changes, since the differences between internal standards and endogenous metabolites were relatively high (RSD 4% and 9%, respectively) (Additional file 1: Table S1). A limitation of this and other metabolomic studies of this type is that the documented metabolite changes are qualitative rather than quantitative, which limits the interpretation of the results more generally. In addition to larger sample sizes and demographics more reflective of the population, future studies may provide benefit by validating and enhancing the biochemical findings of this metabolomic analysis, such as the effect of PLA2 activity and the potential impact of FF/VI on protease activity. Further studies which biochemically compare other treatments to define the overall degree of systemic impact would also be of clinical benefit. The main strength of this analysis is that it provides a detailed assessment of the systemic effects of FF/VI in a small cross-over study, whereas a conventional approach to measure systemic effects would require larger and longer studies to monitor the effects on target organs.

## Conclusions

Metabolomic analysis of FF/VI in 26 adult participants with asthma revealed small changes associated with asthma pathogenesis and inflammation. Overall, biochemical differences between placebo and treatment were subtle, suggesting that the majority of the drug effect was confined to topical activity within the airways. The long duration of topical anti-inflammatory action of FF/VI based on exhaled NO suppression was associated with minor systemic anti-inflammatory changes in the global metabolome. There was also little evidence of significant systemic bioactivity on endocrine, metabolic or other known pathways of glucocorticoid action in the global metabolome after 14 days of treatment with FF/VI.

## Supplementary Information

**Additional file 1:** Supplementary methods and results.

**Additional file 2:** Supplementary data.

## Data Availability

Within 6 months of this publication, anonymised individual participant data, the annotated case report form, protocol, reporting and analysis plan, data set specifications, raw dataset, analysis-ready dataset, and clinical study report will be available for research proposals approved by an independent review committee. Proposals should be submitted to www.clinicalstudydatarequest.com. A data access agreement will be required.

## Abbreviations

Acetyl-CoA: Acetyl coenzyme A
Acyl-CoA: Acyl coenzyme A
AE: Adverse event
ANOVA: Analysis of variance
BHBA: 3-Hydroxybutyrate
BMI: Body mass index
CLP: Complex lipid panel
Comp.: Component
DAG: Diacylglycerol
FeNO: Fraction of exhaled nitric oxide
FEV_1_: Forced expiratory volume in 1 s
FF: Fluticasone furoate
HMG-CoA: 3-Hydroxy-3-methyl-glutarate coenzyme A
ICS: Inhaled corticosteroids
LABA: Long-acting β_2_-agonist
LPC: Lysophosphatidylcholines
LPE: Lysophosphatidylethanolamines
NO: Nitric oxide
PC: Phosphatidylcholine
PCA: Principal component analysis
PE: Phosphatidylethanolamine
PEF: Peak expiratory flow
PLA2: Phospholipase A2
PPB: Parts per billion
RF: Random forest
RSD: Relative standard deviation
SABA: Short-acting β_2_-agonist
SD: Standard deviation
Succinyl-CoA: Succinyl coenzyme A
T1: Baseline
T2: Day 15
T3: Day 21
TAG: Triacylglycerol
TCA: Tricarboxylic acid cycle
VI: Vilanterol trifenatate
