# Supplementary material for: Metabolomic changes related to airway inflammation, asthma pathogenesis and systemic activity following inhaled fluticasone furoate/vilanterol: a randomized controlled trial
Source: Respir Res. 2022 Sep 20;23:258. doi: 10.1186/s12931-022-02164-w (PMC9487108; doi:10.1186/s12931-022-02164-w)
Supplement: Supplementary file 1 — Additional file 1: Supplementary methods and results. [file 12931_2022_2164_MOESM1_ESM.docx]

## Additional file 1

## Supplementary methods

### Sample preparation

Samples were extracted with methanol under vigorous shaking with 2 minutes to precipitate protein and dissociate small molecules bound to protein or trapped in the precipitated protein matric. Samples were then centrifuged to recover chemically diverse metabolites. The resulting extract was divided into five fractions: two for analysis by two separate reverse phase (RP)/UPLC-MS/MS methods using positive ion mode electrospray ionization (ESI); one for analysis by RP/UPLC-MS/MS using negative ion mode ESI; one for analysis by HILIC/UPLC-MS/MS using negative ion mode ESI; and one reserved for backup. Samples were briefly placed on a TURBOVAP (Zymark) to remove the organic solvent. The samples were then stored overnight under nitrogen before preparation for analysis.

### Quality assurance (QA) and quality control (QC)

Several types of QC samples were analyzed in combination with the experimental samples. These included technical replicate samples spaced among experimental samples which were either derived from a pool of extensively-characterized human plasma or generated by combining a small portion of each non-plasma experimental samples. This ensured that all aspects of the process were operating within specifications. QC samples also included a cocktail of QC standards, carefully chosen so as not to interfere with the measurement of endogenous compounds, spiked into every analyzed sample, which allowed for instrument performance monitoring and aiding with chromatographic alignment. Instrument variability was determined by calculating the median relative standard deviation (RSD) for the standards that were added to each sample prior to the injection into mass spectrometers. Overall process variability was determined by calculating the mean RSD for all endogenous metabolites present in each of the pooled technical replicate samples.

Experimental samples were randomized across the platform run, with QC samples spaced evenly among the injections.

### Ultrahigh performance liquid chromatography-tandem mass spectroscopy (ULPC-MS/MS)

The sample extract was dried then reconstituted in solvents which contain a series of standards at fixed concentrations to ensure injection and chromatography consistency, and are compatible to each of the four methods described below.
(1) Analysis using acidic positive ion conditions, chromatographically optimized for more hydrophilic compounds

The extract was gradient-eluted from a C18 column (Waters UPLC BEH C18-2.1x100 mm, 1.7 µm) using water and methanol, containing 0.05% perfluoropentanoic acid (PFPA) and 0.1% formic acid (FA).

#### (2) Analysis using acidic positive ion conditions, chromatographically optimised for more hydrophobic compounds

The extract was gradient eluted from the C18 column using methanol, acetonitrile, water, 0.05% PFPA and 0.01% FA, and is operated at an overall higher organic content

#### (3) Analysis using basic negative ion optimised conditions using a separate dedicated C18 column

The basic extracts were gradient-eluted from the column using methanol and water, however with 6.5 mM ammonium bicarbonate at pH 8.

#### (4) Analysis via negative ionization

Analysis was performed on the aliquot following elution from a HILIC column (Waters UPLC BEH amide 2.1x150 mm, 1.7 µm) using a gradient consisting of water and acetonitrile with 10 mM ammonium formate, pH 10.8.

### Data extraction and compound identification

Raw data were extracted, peak-identified, and QC processed using Metabolon’s hard- and software. Compounds were identified by comparison to library entries of purified standards or recurrent unknown entities. Metabolon maintains a library based on authenticated standards that contain the retention time/index (RI), mass to charge ratio (*m/z*), and chromatographic data (including MS/MS spectral data) on all molecules present in the library. Furthermore, biochemical identifications are based on three criteria: RI within a narrow RI window of the proposed identification; accurate mass match to the library ±10 ppm; MS/MS forward and reverse scores (based on a comparison of the ions present in the experimental spectrum to ions present in the library entry spectrum). The use of all three data points can be utilised to distinguish and differentiate biochemicals.

### Lipidomics platform

Lipids were extracted from samples in methanol:dichloromethane in the presence of internal standards. The extracts were concentrated under nitrogen and reconstituted in 0.25mL of 10mM ammonium acetate dichloromethane:methanol (50:50). The extracts were transferred to inserts and place in vials for infusion-MS analysis. The samples were analyzed via both positive and negative mode electrospray. Individual lipid species were quantified by taking the peak area ratios of target compounds and their assigned internal standards, then multiplying by the concentration of internal standard added to the sample. Lipid class concentrations were calculated from the sum of all molecule species within a class, and fatty acid compositions were determined by calculating the proportion of each class comprised by individual fatty acids.

## Supplementary results

### Detected metabolites

Please see Additional file 2 for all detected metabolites and associated results.

### Quality control of internal standards

Median RSD, representing instrument variation and total variability for actual experimental samples and quantitation of endogenous metabolites within the samples, indicated that the platform produced data in accordance with process specifications (Additional file 1: Table S1).

## Supplementary table

### Additional File 1: Table S1. QC statistics

| **QC sample (matrix)** | **Median RSD (serum)** |
| --- | --- |
| Internal standard | 4% |
| Endogenous metabolites | 9% |
| Complex lipid panel | 13% |

**Abbreviations:** QC, quality control; RSD, relative standard deviation.
